# Cytogenetics of the Hybridogenetic Frog *Pelophylax grafi* and Its Parental Species *Pelophylax perezi*

**DOI:** 10.1093/gbe/evad215

**Published:** 2023-11-28

**Authors:** Anna Dudzik, Dmitrij Dedukh, Pierre-André Crochet, Beata Rozenblut-Kościsty, Hanna Rybka, Paul Doniol-Valcroze, Lukáš Choleva, Maria Ogielska, Magdalena Chmielewska

**Affiliations:** Amphibian Biology Group, Department of Evolutionary Biology and Conservation of Vertebrates, Faculty of Biological Sciences, University of Wrocław, Wrocław, Poland; Laboratory of Non-Mendelian Evolution, Institute of Animal Physiology and Genetics, Czech Academy of Sciences, Liběchov, Czech Republic; CEFE, CNRS, Univ Montpellier, EPHE, IRD, Montpellier, France; Amphibian Biology Group, Department of Evolutionary Biology and Conservation of Vertebrates, Faculty of Biological Sciences, University of Wrocław, Wrocław, Poland; Amphibian Biology Group, Department of Evolutionary Biology and Conservation of Vertebrates, Faculty of Biological Sciences, University of Wrocław, Wrocław, Poland; CEFE, CNRS, Univ Montpellier, EPHE, IRD, Montpellier, France; Laboratory of Fish Genetics, Institute of Animal Physiology and Genetics, Czech Academy of Sciences, Liběchov, Czech Republic; Department of Biology and Ecology, Faculty of Science, University of Ostrava, Ostrava, Czech Republic; Amphibian Biology Group, Department of Evolutionary Biology and Conservation of Vertebrates, Faculty of Biological Sciences, University of Wrocław, Wrocław, Poland; Amphibian Biology Group, Department of Evolutionary Biology and Conservation of Vertebrates, Faculty of Biological Sciences, University of Wrocław, Wrocław, Poland

**Keywords:** hybridogenesis, karyotype, *Pelophylax grafi*, *Pelophylax perezi*, fluorescent in situ hybridization, comparative genomic hybridization

## Abstract

Hybrid taxa from the genus *Pelophylax* can propagate themselves in a modified way of sexual reproduction called hybridogenesis ensuring the formation of clonal gametes containing the genome of only one parental (host) species. *Pelophylax grafi* from South-Western Europe is a hybrid composed of *P. ridibundus* and *P. perezi* genomes and it lives with a host species *P. perezi* (P-G system). Yet it is unknown, whether non-Mendelian inheritance is fully maintained in such populations. In this study, we characterize *P. perezi* and *P. grafi* somatic karyotypes by using comparative genomic hybridization, genomic in situ hybridization, fluorescent in situ hybridization, and actinomycin D-DAPI. Here, we show the homeology of *P. perezi* and *P. grafi* somatic karyotypes to other *Pelophylax* taxa with 2*n* = 26 and equal contribution of *ridibundus* and *perezi* chromosomes in *P. grafi* which supports F1 hybrid genome constitution as well as a hemiclonal genome inheritance. We show that *ridibundus* chromosomes have larger regions of interstitial (TTAGGG)_n_ repeats flanking the nucleolus organizing region on chromosome no. 10 and a high quantity of AT pairs in the centromeric regions. In *P. perezi*, we found species-specific sequences in metaphase chromosomes and marker structures in lampbrush chromosomes. Pericentromeric *RrS1* repeat sequence was present in *perezi* and *ridibundus* chromosomes, but the blocks were stronger in *ridibundus*. Various cytogenetic techniques applied to the P-G system provide genome discrimination between *ridibundus* and *perezi* chromosomal sets. They could be used in studies of germ-line cells to explain patterns of clonal gametogenesis in *P. grafi* and broaden the knowledge about reproductive strategies in hybrid animals.

**Significance**

In the genus *Pelophylax*, there are interspecies hybrids able to propagate, but their reproductive strategy was studied only in one taxon, the edible frog *P. esculentus*, composed of *P. ridibundus* and *P. lessonae* genomes. To elucidate the inheritance pattern in another hybrid *P. grafi*, containing mixed genomes of *P. ridibundus* and *P. perezi*, we analyzed the chromosomal sets of *P. grafi* and *P. perezi*. Although the chromosome number was consistent with other *Pelophylax* taxa (2*n* = 26), we found differences in genomic arrangements between *ridibundus* and *perezi* chromosomes. This will contribute to novel studies of *P. grafi* gametogenesis, particularly to genomic composition of germ-line cells, which will broaden the knowledge about reproductive strategies in hybridogenetic water frogs.

## Introduction

The Western Palearctic water frogs of the genus *Pelophylax* include three main lineages, the *perezi* lineage, the *lessonae* lineage, and the *ridibundus*/*bedriagae* lineage (Lymberakis et al. 2007; Akın et al. 2010). Some of these taxa do hybridize naturally, sometimes resulting in nuclear and mitochondrial DNA introgressions (Kolenda et al. 2017) and giving rise to various mixed populations composed usually of one parental (or host) species and the hybrid with hybridogenetic reproduction, which is also categorized as asexual and non-Mendelian that is without recombination (Plötner et al. 2008; Hoffmann et al. 2015; Dufresnes et al. 2017; Papežík et al. 2021). Hybridogenetic *P. esculentus*, which is characterized by a widespread continental distribution, is originally a hybrid between *P. lessonae* and *P. ridibundus*, living together either with parental *P. lessonae* (the L-E system) or parental *P. ridibundus* (the R-E system) (Berger 1973; Uzzell and Berger 1975; Graf and Polls-Pelaz 1989; Plötner 2005). Peninsular Italy and Sicily are inhabited by hybridogenetic *P. hispanicus*, a hybrid with genomes from *P. bergeri* and *P. ridibundus*, forming a mixed population with parental *P. bergeri* (the I-RI system) (Graf and Polls-Pelaz 1989). Finally, *P. grafi* is considered another hybridogenetic taxon composed of *P. ridibundus* and *P. perezi* genomes. Its distribution ranges from South-Western France to North-Eastern Spain, where it lives with a host species *P. perezi* (the P-G system) (Graf and Polls-Pelaz 1989; Crochet et al. 1995; Sánchez-Montes et al. 2016). Mechanisms of inheritance pathways in the P-G system, presumably analogous to the L-E system (Graf and Polls-Pelaz 1989), remain poorly understood.

Hybrids form clonal gametes in two main steps through a process called hybridogenesis. The first step includes eliminating one of the parental genomes, followed by duplication of the second, non-eliminated parental genome. Elimination of one of the genomes from the germ-line cells of *P. esculentus* hybrids occurs in both sexes before meiosis during very early gametogenesis in specialized gamete ancestor cells that is gonocytes. The elimination is a unique process that takes place during gonocyte interphase when the chromatin of one set of chromosomes is specifically marked by an unknown molecular mechanism and is discarded from the nucleus in the form of micronuclei (Ogielska 1994; Chmielewska et al. 2018). The second mechanism of genome elimination involves micronuclei formation from lagging chromosomes during gonocytes division (Dedukh et al. 2020). Chromatin in micronuclei is highly condensed and becomes transcriptionally inactive and degraded via autophagy (Chmielewska et al. 2018). Using genomic in situ hybridization (GISH), fluorescent in situ hybridization (FISH), or other species-specific cytogenetic markers (Dedukh et al. 2020; Chmielewska et al. 2022), we can trace which genome is eliminated. The remaining chromosomal set is duplicated and chromosomes undergo meiosis, resulting in genetically non-recombined gametes (reviewed by Graf and Polls-Pelaz 1989; Ogielska 2009).

Clonal transmission of non-eliminated genomes from generation to generation requires breeding between a hybrid and a parental species whose genome has been eliminated. Therefore, hybrids typically have to coexist with one of the parental species (Graf and Polls-Pelaz 1989; Plötner 2005). Studies on populations that included *P. esculentus* revealed a high diversity of gametogenic pathways in this hybrid (Dedukh et al. 2015, 2019; Doležálková-Kaštánková et al. 2021; Chmielewska et al. 2022; Pustovalova et al. 2022), whereas *P. grafi* hybrids are currently thought to form only one population type with unknown pathways of gamete formation.

Gametogenesis and genome elimination in *P. esculentus* have generated a lot of scientific interest, as exceptions to the rules are often excellent opportunities to learn about the functioning of near-universal mechanisms such as gametogenesis in vertebrates. In this context, having another model of genome exclusion during gametogenesis would allow testing for the generality of the results obtained in *P. esculentus*. Comparative analysis of karyotypes from two hybridogenetic systems, *P. esculentus* and *P. grafi*, may reveal the strength of the interspecies meiotic barrier preventing genomic admixture, whether karyotypes are conserved or not in the *Pelophylax* genus, and whether the mechanism of genome inheritance in *grafi* are similar to other *Pelophylax*. In addition, because *P. grafi* is currently assumed to persist in a single system (the P-G system where it reproduces with *P. perezi*), one may assume that the hybridogenesis process may be less variable in this organism compared to the widely studied *P. esculentus*. Last, very little is known about ploidy variation in populations of the P-G systems. Schmeller et al. (2001) reported the occurrence of triploids in *grafi* but do not provide the corresponding data. As this has important implications for the biology of the P-G system, assessing ploidy in individuals of the hybrid taxon of the P-G system would provide crucial information for its ecology and conservation.

To investigate the mechanisms underlying genome elimination during hybrid gametogenesis, it is crucial to properly identify parental chromosomes in germ cells. Parental species of the well-studied *P. esculentus*, *P. ridibundus*, and *P. lessonae* (2*n* = 26), do have five large and eight small chromosomes, by which large chromosomes were considered metacentric or submetacentric, while some small chromosomes were also acrocentric (Ebendal 1977; Koref-Santibáñez and Günther 1980; Zaleśna et al. 2011) (table 1). Distinguishing the chromosomes of the parental species in karyotypes of *P. esculentus* hybrids became possible only when Heppich et al. (1982) discovered the difference in the content of centromeric AT repeats. After actinomycin D-DAPI (AMD-DAPI) staining the strong fluorescent signals were detected in centromeres of chromosomes belonging to *P. ridibundus* but not in those of *P. lessonae* (Heppich et al. 1982; Tunner and Heppich-Tunner 1991; Ogielska et al. 2004). Species within *ridibundus* and *lessonae* lineages differ in the number of pericentromeric *RrS1* repeat, which is abundant in chromosomes of the species within the *ridibundus* lineage while less represented in *P. lessonae* chromosomes (Ragghianti et al. 1995; Marracci et al. 2011). The FISH with the probe against *RrS1* repeat allows identifying five large and one small (Ragghianti et al. 1995, 2007; Marracci et al. 2011; Chmielewska et al. 2022) or all *P. ridibundus* chromosomes (Dedukh et al. 2019, 2020). DNA sequence homologous to *RrS1* in *P. lessonae* is *les177.1* (Marracci et al. 2011). A new FISH marker specific for *P. lessonae* genome is the minisatellite sequence *PlesSat01-48* (44 bp), which is present on pericentromeric regions of two chromosome pairs, the acrocentric chromosome 8 and the chromosome 10 (Choleva et al. 2023, preprint). Variability of the interstitial telomeric repeats (ITS) which are flanking the nucleolus organizing region (NOR) on chromosome 10 allows for the recognition of *P. ridibundus* lampbrush and mitotic chromosomes with two ITS on both sides of the NOR from *P. lessonae* chromosomes with one ITS on the distal side of the NOR (Dedukh et al. 2013, 2019). Comparative genomic hybridization (CGH) and GISH successfully identified both parental genomes in *P. esculentus* hybrid cells (Zaleśna et al. 2011; Doležálková et al. 2016). The latter two cytogenetic methods can additionally discriminate large-scale chromosomal rearrangements in hybrids (Bi et al. 2007). Finally, genome composition in oocytes can be identified through the investigation of lampbrush chromosomes isolated from diplotene oocyte nuclei due to their enormous size and distinct morphology (Zlotina et al. 2017). Variation in morphology allows the identification of individual chromosomes and has been successfully used for the discrimination of genomes transmitted by *P. esculentus* females (Bucci et al. 1990; Dedukh et al. 2013, 2015, 2017).

**Table 1:** The karyotype characteristics for chromosomal sets (2N) of *P. ridibundus*, *P. lessonae*, *P. esculentus*, *P. perezi*, and *P. grafi* in accordance with the publications referred to in the text (Ebendal 1977; Koref-Santibáñez and Günther 1980; Zaleśna et al. 2011; Martirosian and Stepanyan 2009) and our results.

Taxon	Chromosome type (2N)						Localization	Source
	Metacentric		Submetacentric		Acrocentric			
	Big	Small	Big	Small	Big	Small		
*Pelophylax ridibundus*	6	6	2^a^	6	0	4^a^	East Germany	Koref-Santibáñez and Günther (1980)
	4	6	6	6	0	4	Armenia	Martirosian and Stepanyan (2009)
	6	6	4	6	0	4	Ejmiatsin, Armenia	Martirosian and Stepanyan (2009)
*Pelophylax lessonae*	8	6	2	6	0	4	N. Uppland in C. Sweden	Ebendal (1977)
	8	8	2	8	0	0	East Germany	Koref-Santibáñez and Günther (1980)
*Pelophylax esculentus*	8	6	2	8 + 2^a^	0	0	East Germany	Koref-Santibáñez and Günther (1980)
	10 Big, 16 Small^b^				0	0	Horní Bludovice in Czech Republic, Poland	Zaleśna et al. (2011)
*Pelophylax perezi*	4	4	6	4	0	8	South-Western France	This study
*Pelophylax grafi*	4	4	6	4	0	8	South-Western France	This study

^a^Refers to chromosomes that were assigned to mixed type (metacentric/submetacentric, submetacentric/acrocentric) in the original publications. Each time we decided to segregate them to the second type.

^b^Authors did not specify which chromosomes are assigned to which types in the original publication.

In this study, we focused on the P-G system involving the parental species *P. perezi* and *P. grafi*, a taxon of hybrid origin carrying one set of *ridibundus* and one set of *perezi* chromosomes (Crochet et al. 1995; Sánchez-Montes et al. 2016). The origin of the P-G system is currently unknown as *P. perezi* and *P. ridibundus* are considered to have been allopatric before current human-induced species translocations; a hypothesis is that the P-G system emerged from crosses between *P. perezi* and *P. esculentus* in Western France where they are sympatric (Dubey and Dufresnes 2017; Dufresnes et al. 2017; Dufresnes and Mazepa 2020). As such, *P. ridibundus* individuals are absent from this system and its genome is passed down from one generation of hybrids to another only through gametes produced by *P. grafi*. One of the difficulties in studies of the P-G system is that *P. ridibundus* and *P. perezi* have very similar morphology, rendering the identification of the hybrid taxon *grafi* currently impossible based on morphology. Since *P. ridibundus* has recently invaded large parts of the range of the P-G system (Demay et al. 2023), safe identification of these three taxa relies on genetic analyses (Cuevas et al. 2022); this complicates considerably targeted sampling of *grafi* and *perezi* individuals in natural populations.

Particularly, we aimed to characterize *P. perezi* karyotypes in both somatic cells and meiotic diplotene oocytes to assess their relationship with other taxa of the *Pelophylax* genus. We also wanted to explore whether chromosomal complements of *P. grafi* hybrids are similar to other *Pelophylax* karyotypes and maintain the integrity of the parental genomes, which may support the hypothesis of clonal gamete production by this hybrid. Finally, we performed a comparison of *P. perezi* and *P. ridibundus* chromosomal features in the somatic karyotypes of *P. grafi* using cytogenetic techniques previously successfully applied on the *P. esculentus* chromosomes. Knowledge of chromosomal features within the P-G system will help us understand inheritance patterns in this system, facilitating the examination of DNA elimination processes in germ-line cells.

## Results

### *Pelophylax perezi* Karyotype

**Fig. 1.**
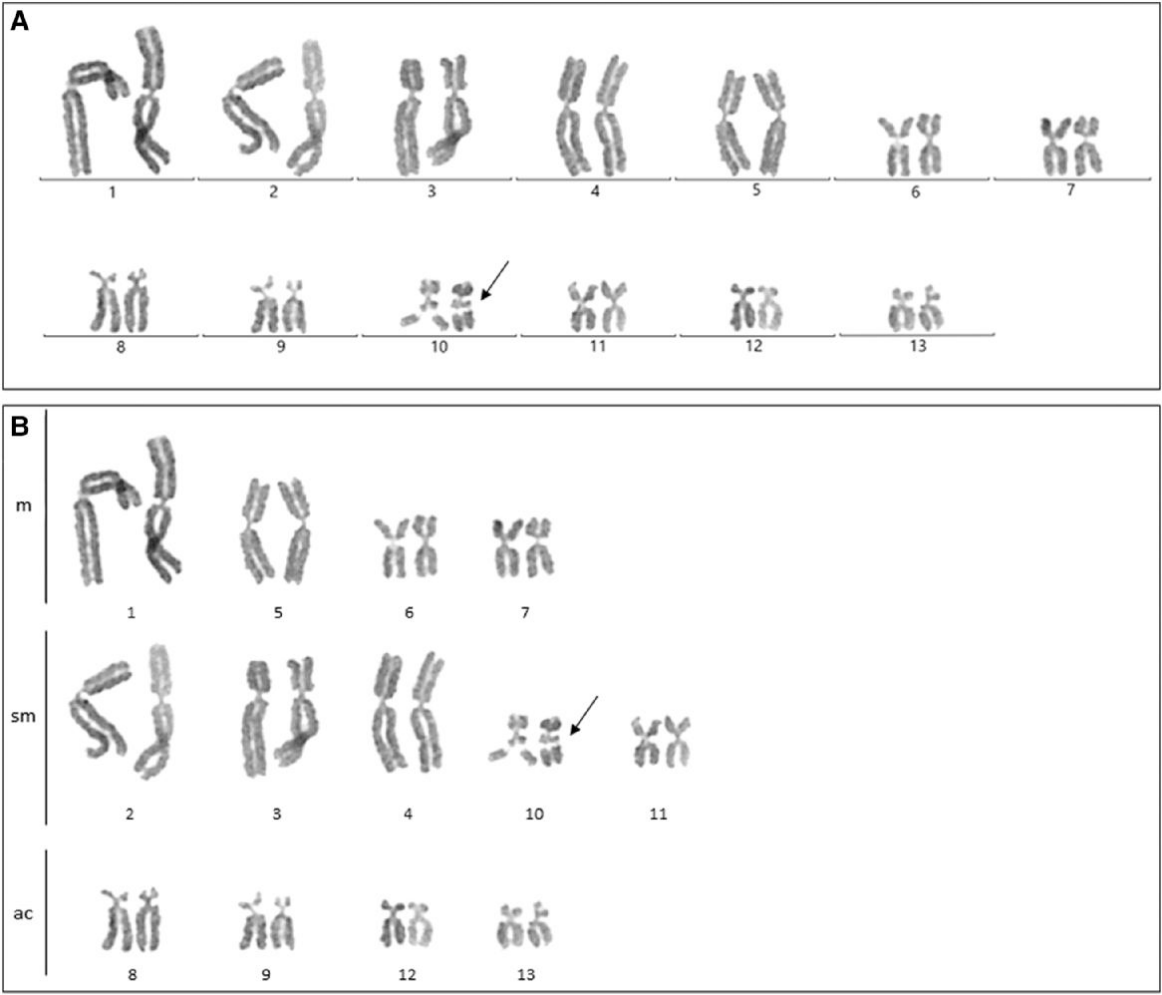
Karyotype of *P. perezi*. Chromosomal spreads were stained with Giemsa. (*A*) The complete diploid genome of *P. perezi* consists of 13 pairs of chromosomes. The pairs are arranged from the largest to smallest, resulting in two groups: five large and eight small. (*B*) Chromosome pairs are divided into classes according to the position of the centromere. Arrows indicate the NOR on chromosome pair no. 10. m, metacentric; sm, submetacentric; ac, acrocentric chromosomes.

*Somatic tissue*. The diploid karyotype of *P. perezi* assessed in two individuals contained 26 chromosomes and was consistent in the number of chromosomes and in their morphology. The karyotype included five pairs of large and eight pairs of small chromosomes, all pairs gradually decreasing in length (fig. 1*A*). The pairs of chromosomes were sorted according to centromere position and divided into the following classes: metacentric (nos. 1, 5, 6, 7), submetacentric (nos. 2, 3, 4, 10, 11), and acrocentric (nos. 8, 9, 12, 13) (fig. 1*B*). Chromosome pairs nos. 8 and 9, as well as 12 and 13 were similar in centromere position but different in length. The NOR was located on the long arm of each chromosome no. 10 (fig. 1—black arrow).

*Lampbrush chromosomes.* We isolated complete 24 lampbrush chromosomal sets from two *P. perezi* females. The lampbrush karyotype of *P. perezi* included 13 fully paired bivalents with clearly distinguishable five large and eight small bivalents (supplementary fig. S1*A*, Supplementary Material online). Centromeric regions were indistinguishable neither under the phase contrast microscope nor after DAPI staining. The chromosome arrangement was performed after FISH with the *RrS1* probe detecting pericentromeric heterochromatin (supplementary fig. S1*B*, Supplementary Material online). Using FISH with an oligonucleotide probe specific to (TTAGGG)_n_ repeat, we detected interstitial telomeric sites (ITS) (supplementary fig. S2, Supplementary Material online). Additionally, we evaluated the distribution of marker structures along the chromosomal axis, including chromosomes-associated spheres and nucleoli, and loops with unusual morphology and an intense accumulation of ribonucleoprotein matrix, distinguishing them from other lateral loops. However, the precise recognition of certain marker loops was challenging due to the condensed structure of lateral loops. Still, it allowed us to construct the cytological maps of lampbrush chromosomes where the relative position of the most prominent marker structures was indicated (supplementary fig. S3, Supplementary Material online). Lampbrush chromosomes were arranged by size and numbered with the letters (A–M) as they may differ in comparative length with somatic metaphase chromosomes due to various levels of chromatin condensation.

Further, we identified the relative position of all prominent marker structures on *P. perezi* lampbrush chromosomes (supplementary figs. S1 and S3, Supplementary Material online). Chromosome A is the longest one at the lampbrush stage. In the subtelomeric regions of both arms, we identified noticeable marker loops (supplementary figs. S1 and S3, Supplementary Material online). The long arm of B had a sphere in its subtelomeric region and a marker loop in the pericentromeric region of the short arm (supplementary figs. S1 and S3, Supplementary Material online). In the long arm of C, we detected long marker loops and lumpy loops (supplementary figs. S1 and S3, Supplementary Material online). Chromosomes D and E have long marker loops in the short arms (supplementary figs. S1 and S3, Supplementary Material online), F does not have prominent marker structures (supplementary figs. S1 and S3, Supplementary Material online), G has a marker loop in the pericentromeric region of the short arm (supplementary figs. S1 and S3, Supplementary Material online), and H has a lumpy loop, marker loop, and active NOR locus with attached nucleoli (supplementary figs. S1 and S3, Supplementary Material online). Furthermore, FISH with an oligonucleotide probe specific to (TTAGGG)_n_ repeat revealed three small interstitial blocks of this repeat on chromosome H (supplementary fig. S2, Supplementary Material online). On the long arm of J, we found two marker loops (supplementary figs. S1 and S3, Supplementary Material online) and a sphere in the short arm, as well as a marker loop and a lumpy loop in the long arm (supplementary figs. S1 and S3, Supplementary Material online). On the long arms of K and L, we detected marker loops in the long arms (supplementary figs. S1 and S3, Supplementary Material online). Chromosome M had a sphere in the short arm and two pairs of marker loops in the long arm (supplementary figs. S1 and S3, Supplementary Material online).

### *Pelophylax grafi* karyotype

**Fig. 2.**
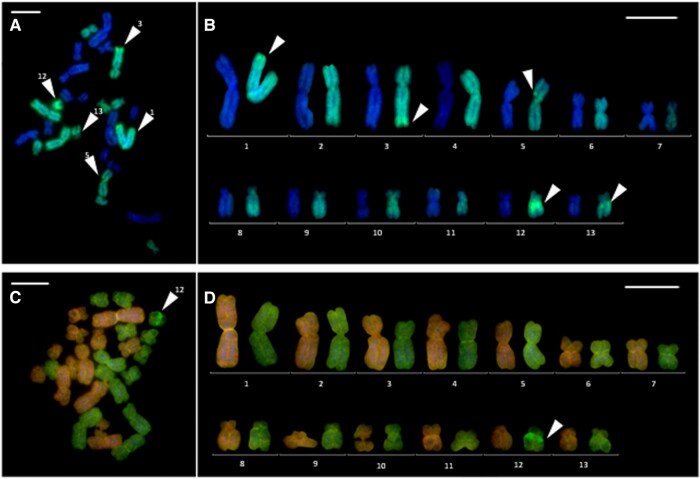
Karyotype of *P. grafi*. GISH (*A*, *B*) and CGH (*C*, *D*) on chromosomes with the whole-genomic probe from *P. perezi* (*A*, *B*) or the mixture of two whole-genomic probes from *P. perezi* and *P. ridibundus* (*C*, *D*) counterstained with DAPI. (*A*) Metaphase plate and (*B*) karyotype containing 26 chromosomes: 13 belonging to *P. ridibundus* (DAPI—chromosome on the left side of the pair) and 13 to *P. perezi* (AlexaFluor488—chromosome on the right side of the pair); (*C*) metaphase plate and karyotype containing 26 chromosomes: 13 belonging to *P. ridibundus* (RhodamineRed—chromosome on the left side of the pair), 13 to *P. perezi* (AlexaFluor488—chromosome on the right side of the pair). Arrowheads indicate possible species-specific sequences on *P. perezi* chromosomes. Scale bars 10 µm.

*Somatic tissue.* The diploid karyotype (2*n* = 26 chromosomes) of *P. grafi* was assessed in eight individuals. All chromosomes showed strong hybridization, which indicated that the *P. grafi* karyotype consists of 13 *P. ridibundus* and 13 *P. perezi* chromosomes, clearly seen both from GISH (fig. 2*A* and *B*) and CGH (fig. 2*C* and *D*) analyses. The pairs of chromosomes were metacentric, submetacentric, and acrocentric with pair no. 10 bearing the NORs, as in the case of the *P. perezi* karyotype. Using the GISH technique with the *P. perezi* whole-genomic probe and blocking cot10 DNA from *P. ridibundus,* we distinguished species-specific *P. perezi* heterochromatin on the chromosomes from pairs nos. 1, 3, 5, 12, 13 (fig. 2*A* and *B*—white arrowheads), while chromosomes nos. 1 and 3 showed stronger signals in subtelomeric regions (on *p* or *q* arms, respectively). *Pelophylax perezi* chromosomes from pairs nos. 5, 12, and 13 showed stronger signals in the pericentromeric regions; no. 5 showed stronger signals on the *p* arms, and nos. 12 and 13 showed stronger signals on the *q* arms. The signal of the pericentromeric region on chromosome pair no. 12 always appeared as the most intensive and remained highly visible even in CGH-treated metaphase plates (fig. 2*C* and *D*—white arrowhead). In *P. grafi* karyotypes, *P. ridibundus* chromosomes had a similar size as compared to *P. perez*i. Neither GISH nor CGH-treated metaphase chromosomes showed genome introgressions within a chromosomal structure. None of the analyzed metaphase plates indicated the existence of polyploid individuals.

### Pericentromeric Heterochromatin and Telomeric (TTAGGG)_n_ Repeats in *P. perezi* and *P. ridibundus* Chromosomes

**Fig. 3.**
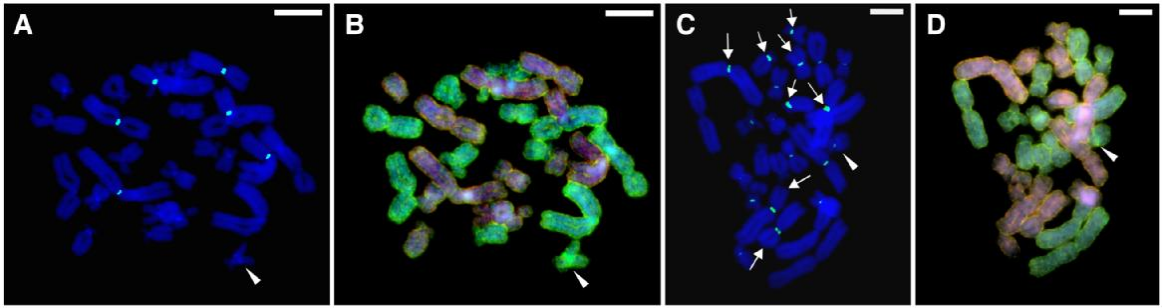
Comparison of the abundance of *RrS1* pericentromeric regions between *perezi* and *ridibundus* chromosomes in the karyotypes of *P. grafi* hybrid. Sequential hybridization of two metaphase plates (*A*–*B* and *C*–*D*): (*B*, *D*) first step*—*CGH with the mixture of two whole-genomic probes from *P. perezi* (AlexaFluor488—marked with arrowheads) and *P. ridibundus* (RhodamineRed) to affiliate chromosomes to parental genome type, (*A*, *C*) second step*—*wash of the probes and FISH with the probe to pericentromeric *RrS1* repeat (pericentromeric dot signals), counterstained with DAPI. (*A*, *C*) Artificially adjusted threshold for RrS1 probe signal allowed to distinguish (*A*) six chromosomes with the strongest *RrS1* signals, which belong to *P. ridibundus*, or (*C*) eight chromosomes with the highest *RrS1* signals (arrows), which belong to *P. ridibundus*. The arrowhead indicates possible AT-rich sequences on chromosome no. 12 belonging to *P. perezi*. Note that in (*C*) some *P. perezi* chromosomes still have visible *RrS1* signals, which makes the distinction of genome type impossible. Scale bars 10 µm.

**Fig. 4.**
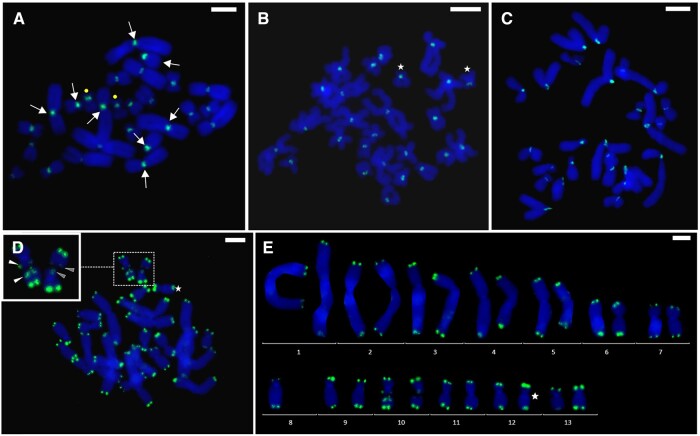
Pericentromeric *RrS1* regions and interstitial telomeric regions in the chromosomes of the hybrid *P. grafi* and their parental species *P. perezi* and *P. ridibundus.* FISH with *RrS1* probe on (*A*) *P. grafi*, (*B*) *P. perezi*, and (*C*) *P. ridibundus* chromosomes. Although *P. grafi* chromosomes are characterized by visually distinctive intensities of *RrS1* signal on eight chromosomes (arrows), the differences are insufficient to assess the species-specific affiliation of individual chromosomes. Chromosomes no. 10 are marked with dots (*A*), *P. perezi* chromosomes no. 12 with visible heterochromatin blocks are depicted by an asterisk (*B*, *D*, *E*). (*D*, *E*) FISH on telomeric sequences on *P. grafi* chromosomes using (TTAGGG)_n_ telomeric probe reveals interstitial sequences on chromosome pair no. 10 enlarged in the inset. *Pelophylax ridibundus* chromosome on the left has more prominent staining of interstitial sequences (arrowheads) than *P. perezi* chromosome on the right (darker arrowheads). (*E*) Karyogram of the chromosomes presented in (*D*). One of the chromosomes from pair no. 8 is lacking in the metaphase plate. Scale bars 10 µm.

FISH with a pericentromeric probe to the *RrS1* repeat, specific to the *P. ridibundus* genome, was performed on *P. ridibundus*, *P. perezi*, and the hybrid *P. grafi* individuals and showed that the probe efficiently hybridized with both *P. ridibundus* and *P. perezi* chromosomes (fig. 4*A*–*C*). The fluorescence level of the signal intensity differed between the genomes of the two species and was higher in the *ridibundus* chromosomes, visualized by performing FISH on slides previously used for the CGH (sequential staining). Artificially lowered level of brightness for green color signals of the probe to *RrS1* repeat on *P. grafi* karyotypes revealed that on several figures, the signals on five large and one small *ridibundus* chromosomes were the most prominent, while on the other *P. ridibundus* and all *P. perezi* chromosomes, the signal was weak or invisible (fig. 3*A* and *B*). Using solely the FISH technique, it was rather difficult to differentiate parental sets in the hybrids’ tissues, however, some chromosomes had higher proficiency in combining with the probe to *RrS1* repeat (fig. 4*A*, white arrows). The comparison with FISH-treated metaphase plates obtained from *P. perezi* somatic tissues shows that pericentromeric fluorescence signals were lower and rather uniform than those of *P. grafi* and *P. ridibundus* (fig. 4*A*–*C*). Adjusting the threshold for the green color allowed us to distinguish eight chromosomes belonging to *P. ridibundus* (fig. 3*C* and *D*). Additionally, we observed that the species-specific sequence on chromosome 12 of *P. perezi* genome is possibly AT-rich, due to the strong DAPI signal in this area (fig. 3*A*–*D*, white arrowheads).

FISH with an oligonucleotide probe specific to telomeric repeats (TTAGGG)_n_ performed on *P. grafi* revealed interstitial telomeric sequences on both chromosomes no. 10 (fig. 4*D*—arrowheads, *E*), which have secondary constrictions (NOR) on the long arm visible as dark areas on the proximal part of the q-arm. Hybrid metaphase plates showed that interstitial sequences flanked both sides of the NOR region of the *ridibundus* chromosome no. 10 (fig. 4*D*—white arrowheads). Although *P. perezi* genome showed ambiguous results, it was visible that NORs were flanked from both sides with interstitial (TTAGGG)_n_ sequences yet less abundant (fig. 4*D*—gray arrowheads), as reflected by lower staining intensity than those of *P. ridibundus*. This subtle difference in NOR staining was sufficient enough to distinguish *ridibundus* and *perezi* chromosomes in the *P. grafi* genome (fig. 4*D* and *E*).

AMD-DAPI performed on *P. ridibundus* chromosomes showed quite distinctive bright spots in the centromeric regions (supplementary fig. S4*A*, Supplementary Material online) indicating AT-rich heterochromatin, while *P. perezi* chromosomes were more homogenous (supplementary fig. S4*B*, Supplementary Material online), and the centromeric regions stained weakly and gave faint signals. The difference in the signal intensity is well visible in metaphase plates of the *P. grafi* hybrid (supplementary fig. S4*C*, Supplementary Material online).

## Discussion

### *Pelophylax perezi* and *P. grafi* Somatic Karyotypes

**Fig. 5.**
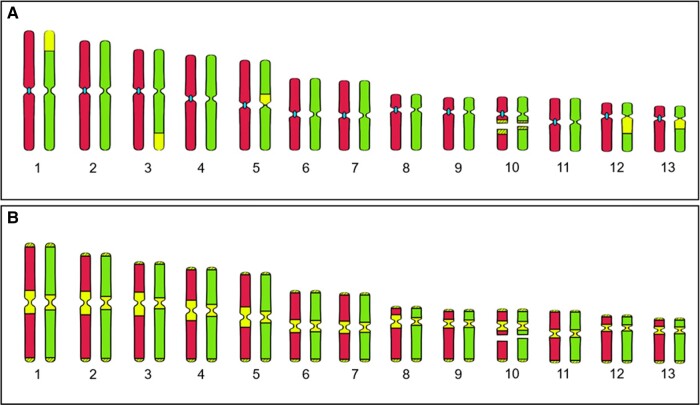
Diagrams summarizing (*A*) differences and (*B*) similarities between two parental genomes, *P. ridibundus* (chromosome on the left side of the pair) and *P. perezi* (chromosome on the right side of the pair), examined in a hybrid *P. grafi*. (*A*) Centromeric ovals represent AT-rich centromeres present on *P. ridibundus* chromosomes, *P. perezi* species-specific sequences present on chromosomes nos. 1, 3, 5, 12, and 13 are marked with different color. Areas with diagonal stripes represent interstitial (TTAGGG)_n_ repeats, more pronounced on the *P. ridibundus* chromosome no. 10. (*B*) Chromosome areas with diagonal stripes depicted on chromosomal ends are telomeres, marked pericentromeric areas are *RrS1* pericentromeric repeats, more abundant on eight *P. ridibundus* chromosomes (five large ones and three small ones, supposedly nos. 6, 7, and 8).

Karyotypes of *P. perezi* and its hybrid *P. grafi* consisted of 13 pairs of chromosomes (2*n* = 26). The studied *P. grafi* had integral parental chromosomal sets and maintained F1 hybrid genome constitution (fig. 2, fig. 5*A* and *B*) without visual blocks of homologous recombination. Comparative analyses of karyotypes between *P. perezi* (this study) and previously studied *P. ridibundus*, *P. lessonae*, and hybrid *P. esculentus* (Koref-Santibáñez and Günther 1980; Zaleśna et al. 2011) showed vast similarities in chromosome morphology (table 1). Diploid karyotypes of *P. perezi* and *P. grafi* were composed of five pairs of large and eight pairs of small chromosomes, as in other *Pelophylax* taxa studied so far. We characterized *P. perezi* karyotype as with eight metacentric, ten submetacentric, and eight acrocentric chromosomes (fig. 1 and table 1). The choosing of categories to which the chromosomes were assigned was conducted based on visual comparisons with existing literature and researchers’ experience concerning *Pelophylax* karyotypes. Measure-based categorization was deemed insufficient due to the impact of the methodology on the morphology of chromosomes (Heppich 1978; Hnátková et al. 2023; Krysanov et al. 2023). The occurrence of eight acrocentric chromosomes is not a common phenomenon in water frogs and was reported only in *P. perezi* (this study). In earlier reports, the numbers of acrocentrics ranged from four to zero (Ebendal 1977; Koref-Santibáñez and Günther 1980; Martirosian and Stepanyan 2009; Zaleśna et al. 2011), suggesting diversity at the population level. However, it is important to mention that those results were obtained largely during chromosomal measurements. Overall, the karyotype of *P. perezi* and other *Pelophylax* species is generally stable, rarely it shows even extended variation. It is supported by an evolutionary context of a single ancestor of the Western Palearctic water frogs *Pelophylax* revealed by the mitochondrial phylogeography studies (Lymberakis et al. 2007; Dufresnes and Mazepa 2020), as well as karyotype studies in which the results were superimposable (table 1). Despite the above-mentioned data in Bucci et al. (1990), it is reported that in samples from Central Poland, chromosome pair no. 11 was metacentric in *P. ridibundus* and *P. lessonae,* and chromosome pair no. 12 was submetacentric in *P. ridibundus* and metacentric in *P. lessonae* (Bucci et al. 1990). We do not know yet whether this variation has an evolutionary context or it simply comes from a different methodology. As an example, *P. lessonae* from France had submetacentric and metacentric chromosome pair no. 11 caused by the centric inversion (discussed in Tunner and Heppich 1983). All 15 *P. grafi* individuals studied here were diploid, whereas Schmeller et al. (2001) reported the occurrence of 20 triploids in a sample of 234 individuals of this taxon. Further data on the ploidy of *grafi* individuals are needed to understand if triploids occur at low frequency in most populations of the P-G system or if they reach high proportions in some populations, as is observed in the L-E system.

### GISH and CGH in the Hybrid *P. grafi*

Applying the GISH technique with *P. perezi* whole-genomic probe on *P. grafi*, we demonstrated that *P. perezi* chromosomes exhibited several species-specific sequences schematically presented in figure 5*A* and *B*. Both methods clearly visualized two distinct groups of chromosomes and differentiated chromosomes belonging to *P. ridibundus* from those belonging to *P. perezi*. We confirmed the genomic integrity of the two parental chromosome sets in somatic cells of *P. grafi,* which indirectly supports functional hybridogenetic mechanisms of clonal reproduction in these taxon, efficiently restricting gene flow between parental genomes as known from *P. esculentus* (Zaleśna et al. 2011) or clonal fishes (Majtánová et al. 2016, 2021).

The *ridibundus* and *perezi* chromosomes were similar in size when we compared 13 pairs of homologs on the karyograms of *P. grafi*; yet, *perezi* chromosomes seem to be slightly smaller. Combining the current data with those presented by Zaleśna et al. (2011), *lessonae* chromosomes studied in hybrid *P. esculentus* were much smaller when compared with *P. ridibundus* and *P. perezi*.

GISH performed with a whole-genomic probe from *P. perezi* and *P. ridibundus* cot10 DNA highlighted heterochromatin blocks that could be tandem repeats on five *perezi* chromosomes, three (nos. 5, 12, and 13) in pericentromeric regions and two (nos. 1 and 3) in telomeric regions. Although the CGH procedure does not predispose to greater specificity of binding by possible occurrence of repeats contained in the whole-genomic probes, the pericentromeric region of chromosome no. 12 remained intensively highlighted throughout nearly all of the metaphase plates studied (fig. 2*A*–*D*) and is a potentially useful species-specific pericentromeric marker for future studies in the P-G system.

GISH and CGH remain highly useful cytogenetic methods used in studies on hybrid and allopolyploid animals. GISH well-distinguished the *ridibundus* and *lessonae* parental chromosomes in the hybrid *P. esculentus* within L-E, R-E, R-E-L, and E-E systems (Zaleśna et al. 2011; Chmielewska et al. 2022). GISH was also successfully applied to hybrid and allopolyploid gynogenetic unisexual salamanders of the *Ambystoma laterale*-*jeffersonianum* complex (Bogart et al. 2007; Bogart and Bi 2013). The CGH technique was effectively implemented in the R-E system (Doležálková et al. 2016), also allowing us to clearly distinguish *perezi* and *ridibundus* genomes in *P. grafi* karyotypes. The difference between GISH and CGH is methodological. In the first case, a genome of one of the parental species is blocked with the whole-genomic DNA or cot10 DNA (whole-genomic DNA enriched with repetitive sequences), which blocks dispersed repeats, and the other is aligned with a species-specific probe, which results in more pronounced signals of species-specific sequences including species-specific heterochromatin blocks (Symonova et al. 2015). In the case of CGH, the repetitive sequences are not drastically removed. Most evenly distributed retrotransposons of both genomes are aligned with a species-specific probe, visualizing the integrity of genomes or genome introgressions in metaphase plates.

### Pericentromeric Heterochromatin and Telomeric (TTAGGG)_n_ Repeats in *P. perezi* and *P. ridibundus* Chromosomes

We detected pericentromeric *RrS1* repeats in *P. grafi* (with *perezi* and *ridibundus* chromosomes) and in *P. perezi*, with a more intense fluorescent signal of the probe on *ridibundus* than *perezi* chromosomes. Our observation might have been connected to likely a higher number of *RrS1*-like repeats in pericentromeric regions of *P. ridibundus* in comparison with *P. perezi* chromosomes.

Interestingly, we did not observe morphologically distinct centromeric regions on *P. perezi* lampbrush chromosomes that would suggest a smaller size of pericentromeric repeats in this species (supplementary fig. S1, Supplementary Material online). Similarly, no centromeres at the lampbrush chromosomal stage were observed in *P. lessonae* from Poland (Bucci et al. 1990). Nevertheless, we visualized centromeric regions on lampbrush chromosomes using FISH with a probe against *RrS1* repeat and its signal on *P. perezi* lampbrush chromosomes seems to be weaker than those obtained from *P. ridibundus* (Dedukh et al. 2013).

Ragghianti et al. (1995) reported that pericentromeric *RrS1* repeats are abundant in *ridibundus* and relatively infrequent in *lessonae* chromosomes (around 2% of the genome). FISH with the *RrS1* probe allowed the identification of either all *ridibundus* chromosomes (Dedukh et al. 2019, 2020, this study) or at least six chromosomes (five large and one small chromosomes no. 8 [Ragghianti et al. 1995, 2007; Marracci et al. 2011; Chmielewska et al. 2022]). The latter pattern seems to be typical for a *ridibundus*/*bedriagae* lineage, considering that Marracci et al. (2011) observed such a signal also from *P*. *kurtmuelleri* and *P. bedriagae*. We observed this pattern on *ridibundus* chromosomes under methodological conditions when FISH was made after CGH, otherwise, the result was ambiguous.

The interstitial telomeric repeats flanked the NOR regions on the chromosome pair no. 10 and the signal was stronger in *ridibundus* than in *perezi*, suggesting differences in the copy number of this repeat. Previously mapping (TTAGGG)_n_ probe on the lampbrush chromosome of *P. ridibundus*, we observed a big cluster of ITS with approximately 8% from the length of the whole lampbrush chromosome H located in the region closer to the centromere (Dedukh et al. 2013). However, on lampbrush chromosomes of *P. perezi*, we found three small ITSs in the regions flanking NOR region (supplementary fig. S2, Supplementary Material online). Both *RrS1* and telomeric repeats also distinguished *P. perezi* from *P. lessonae* chromosomes which did not bind the respective probes (Dedukh et al. 2015). In addition, we did not find ITS in the subtelomeric region of the long arm of lampbrush chromosome B of *P. perezi* (supplementary fig. S2, Supplementary Material online), contrary to *P. ridibundus* chromosomes (Dedukh et al. 2013). ITS are known to form due to chromosomal rearrangements, telomeres fusion of ancestral chromosomes, or after reparation of double-stranded breaks (Lin and Yan 2008). Given the similar location of one small (TTAGGG)_n_ site on *P. perezi* chromosome to *P. ridibundus* and *P. lessonae* chromosomes, we propose that this site originated in their common ancestor. Additionally, *P. perezi* and *P. ridibundus*, but not *P. lessonae*, exhibit an additional (TTAGGG)_n_ site, differing in size, likely resulting from its amplification on the *P. ridibundus* chromosome. The expansion of microsatellites and (TTAGGG)_n_ sequences may be caused by replication errors due to DNA polymerase slippage (Lin and Yan 2008) and was suggested to explain ITS size difference in such loci on human chromosomes (Mondello et al. 2000) However, the origin of this ITS loci on water frogs chromosomes requires further investigations.

Actinomycin D treatment, which is a nonfluorescent DNA intercalator to GC pairs, combined with DAPI staining (AMD-DAPI) allowed us and others to show a visible difference concerning centromeric AT repeats in chromosomes belonging to different species of the genus *Pelophylax* (Heppich et al. 1982; Ogielska et al. 2004). Zaleśna et al. (2011) proved that *P. ridibundus* chromosomes contain higher quantities of such repeats in comparison to those of *P. lessonae*. We have repeated this experiment for the P-G complex and obtained parallel result in which *P. ridibundus* chromosomes contained a higher copy number of these repeats in comparison to those of *P. perezi*. This differentiation seems to be a useful tool to discriminate between the two sets of parental chromosomes in hybrid *P. grafi*.

### *Pelophylax perezi* Lampbrush Chromosomes

Identification of genome composition in oocytes is possible using the analysis of lampbrush chromosomes isolated from diplotene nuclei (Zlotina et al. 2017). The large size and distinct morphology allowed the identification of individual chromosomes which was successfully used for species-specific discrimination of genomes transmitted by females of *P. esculentus* complex (Bucci et al. 1990; Dedukh et al. 2013, 2015, 2017).

Analysis of lampbrush chromosomes isolated from growing oocytes clearly revealed 13 fully paired bivalents (five large and eight of smaller size) corresponded well with the mitotic (somatic) karyotype. These results are similar to those of *P. ridibundus* and *P. lessonae* from various localities (Bucci et al. 1990; Dedukh et al. 2013). Comparison of the chromosome morphology of *P. perezi* (this study) with those of *P. ridibundus* and *P. lessonae* showed four distinct groups of chromosome homology. 1) Lampbrush chromosomes A, C, and J of *P. perezi* have a conservative distribution of marker structures which are similar among all three species. Nevertheless, 2) chromosomes B, E, I, and M of *P. perezi* resemble the corresponding chromosomes of *P. ridibundus*, while 3) chromosomes G, H, and L of *P. perezi* resemble more chromosomes of *P. lessonae*. 4) Only three chromosomes (D, F, and G) of *P. perezi* have a different and generally poorer distribution of marker structures from corresponding *P. ridibundus* and *P. lessonae* chromosomes, which is unique for *P. perezi*. Similar to *P. ridibundus* and in contrast to *P. lessonae*, lampbrush chromosome H of *P. perezi* bears associated nucleoli, suggesting the presence of active NOR during the lampbrush stage. We believe that the observed marker structures on lampbrush chromosomes of *P. perezi* can be used for further recognition of the genome transmitted by *P. grafi* oocytes.

## Conclusions

Somatic karyotypes of *P. perezi* and *P. grafi* provided a new evidence for conserved chromosome morphology in the *Pelophylax* taxa. Genome integrity was shown in both *perezi* and *ridibundus* chromosomes in hybrid *P. grafi* supporting a hemiclonal genome inheritance in this hybrid. FISH to *RrS1* genomic sequence so far successfully used to distinguish *ridibundus* from *lessonae* chromosomes gave ambiguous results for the discrimination between *ridibundus* and *perezi* chromosomes. Future research on the genome elimination mechanism in germ-line cells in *P. grafi* may be performed by applying GISH and CGH together with AMD-DAPI, which were proven to efficiently discriminate both parental genomes. Species-specific sequences revealed in *P. perezi* chromosomes with the whole-genomic probe are a promising lead for the future creation of FISH probes designed for *perezi* genome. Finally, this study revealed another promising marker with a slightly different expression pattern in FISH for telomeric repeats, where *perezi* chromosome no. 10 showed smaller ITS sequences flanking the NOR than in *ridibundus* chromosome. This sequence and variation in marker structures can be used in studies of lampbrush chromosomes in *P. grafi* oocytes to investigate patterns of clonal gametogenesis in *P. grafi*. Comparison of *P. ridibundus*, *P. perezi*, and *P. grafi* genomes in our and other available data clearly show that the *ridibundus* chromosomes do not differ significantly between the hybrids while *lessonae* and *perezi* do. This strongly suggests that the *ridibundus* genome promotes and enforces the elimination of *lessonae* or *perezi* chromosomes from the germ line and therefore is a clue to hybridogenesis.

## Materials and Methods

### Animals

For the genomic probes and cytogenetic study of *P. grafi* and *P. perezi*, we selected 20 animals (4 adults, 11 juveniles and metamorphs, and 5 tadpoles, of which 14 were females, three males, and three non-sexed) (supplementary table S1, Supplementary Material online) collected in 2021 from three mixed *P. perezi*-*P. grafi* (P-G) populations in Southern France. Sixteen individuals collected from the stream below the Salagou dam (43.6575°N, 3.4070°E, WGS84) included 11 *P. grafi* females, 1 *P. perezi* male, and 3 non-sexed *P. grafi*. Another two *P. perezi* males were collected in the Baillaury River (42.4519°N, 3.0857°E) and Creux de Miège pond (43.5230°N, 3.8195°E). Two *P. perezi* and one *P. grafi* female were collected from a small temporary stream near Le Ravaner upstream from Pradells, Argéles-sur-Mer (42.5198°N, 3.0550°E). All individuals were collected in accordance with French legal regulations concerning wild species protection under the permit provided by the Direction regionale de l'environnement, de l'amenagement et du logement d'Occitanie, no. 2021-s-26.

Two *P. ridibundus* males used for AMD-DAPI staining and DNA extractions for genomic probes were collected in Ruda Milicka (51.533153°N, 17.335117°E), Poland, in 2016, following the permission of the Regional Directorate for Environmental Protection in Wrocław no. WPN.6401.177.2016.IL. Lampbrush chromosomes from diplotene oocytes were performed on two *P. perezi* females, collected from Begues (41.2889°N, 1.9119°E) and La Pobla de Benifassà in Spain (40.7072°N, 0.1647°E), approved by the Servei de Fauna i Flora del Departament de Territori i Sostenibilitat de la Generalitat de Catalunya, permit numbers (SF/0040, SF/0041, SF/0042, and SF/0043). All experiments were carried out following National and International guidelines under the permit from the Local Commission for Ethics in Experiments on Animals in Wrocław, Poland, number 27/2016 and 040/2021/P1.

### Species Identification

Taxonomic determination was based on nuclear DNA variants isolated from muscles (GeneMATRIX TISSUE DNA Purification Kit, EURx Ltd., Poland) using restriction fragments length polymorphism PCR (RFLP PCR) targeting species-specific substitutions in the recombination activating protein 1 (*RAG1*) and tyrosinase 1 (*Tyr1*), see Cuevas et al. (2022), with the modification of forward *RAG1* primer Mart-FL1 (5′-ATGTGCAGYCAGTACCACAAAATG-3′) (Chiari et al. 2004, modified). The protocol was carried out with an annealing temperature of 58 °C for *RAG1* performed in a C1000 Thermal Cycler (Bio-Rad, USA) and 54 °C for *Tyr1* performed in Thermal Cycler (Applied Biosystems, USA).

Afterward, PCR products were incubated with restriction enzymes (Thermo Fisher Scientific, USA) for *RAG1*: EcoO1091 (Dra II) (RGzGNCCY) and for *Tyr1*: Bal I (Mls I) (TGGzCCA). Digested PCR products were run in agarose gel electrophoresis in 1.5% agarose, and results were documented using the Molecular Imager GelDoc XR+ system with ImageLab software (Bio-Rad). DNA size marker Perfect 100 bp DNA Ladder (EURx, Poland) was used as a size standard. Results were interpreted according to Cuevas et al. (2022), where EcoO1091/Dra II digested 936 bp *RAG1* PCR product in *P. ridibundus*, which resulted in two fragments, 565 and 371 bp long, and Bal I/Mls I digested 601 bp Tyr1 PCR product in *P. perezi* resulting in two fragments, 318 and 283 bp long.

### Preparation of Chromosomes

Adult and juvenile animals were injected intraperitoneally with 1 ml or 0.5 ml (for animals with a body mass below 30 g) of 0.3% colchicine solution (Sigma-Aldrich) 24 h before dissection. Metamorphs and tadpoles were placed in a beaker with 0.1% colchicine solution overnight.

Shortly before sacrificing, adult and juvenile animals were anesthetized with a 0.5% water solution of MS 222 (Sigma-Aldrich), while metamorphs and tadpoles were treated with a 0.25% water solution of MS 222 (Sigma-Aldrich). A piece of intestine was dissected from each individual, hypnotized in 0.075 M KCl for 10 min, and fixed in Carnoy fixative (ethanol:glacial acetic acid in 3:1 proportion). After three changes of Carnoy fixative, samples were stored at −20 °C until use.

To obtain chromosomal spreads, small pieces of intestine tissue were placed in 70% acetic acid in the glass preparation chamber and gently homogenized using forceps under a stereomicroscope. The cell suspension was then spread over sloped slides placed on a heater set at +60 °C. Afterward, the chromosomes on the slides were stained with the Giemsa solution.

Metaphase plates on slides were initially scanned by the Zeiss Axioplan epifluorescence microscope equipped with a Charge Coupled Device (CCD) camera and ZEISS Axio Imager.Z2 epifluorescence microscope (Zeiss, Oberkochen, Germany) with Metafer platform (MetaSystems, Altlussheim, Germany). Selected slides were afterward de-stained in ethanol series (50%, 70%, 96%) and used for further staining. Classification of chromosome types (metacentric, submetacentric, acrocentric) was performed based on the morphological features and comparisons to existing literature.

### Preparation of Lampbrush Chromosomes

Lampbrush chromosomes were prepared from diplotene oocytes of two *P. perezi* females according to a previously published protocol (Gall et al. 1991). Ovaries from *P. perezi* females were dissected and placed in the OR2 saline (82.5 mM NaCl, 2.5 mM KCl, 1 mM MgCl_2_, 1 mM CaCl_2_,1 mM Na_2_HPO_4_, 5 mM 4-(2-hydroxyethyl)-1-piperazineethanesulfonic acid; pH 7.4). To isolate nuclei, each oocyte was disrupted manually in isolation medium 5:1” (83 mM KCl, 17 mM NaCl, 6.5 mM Na_2_HPO_4_, 3.5 mM KH_2_PO_4_, 1 mM MgCl_2_, 1 mM dithiothreitol; pH 7.0–7.2). Oocyte nuclei were transferred to a 5:1 medium diluted four times with the addition of 0.1% paraformaldehyde (PFA) and 0.01% 1 M MgCl_2_. The nucleus envelope of each nucleus was manually disrupted to isolate nucleoplasm which was transferred to glass chambers attached to a slide and filled with one-fourth strength 5:1 medium. After the spreading of nucleoplasm containing chromosomes in the chamber, slides were centrifuged for 20 min at +4 °C, 4,000 rpm (Hettich universal 320, Germany). Afterward, chromosomes were fixed for 30 min in 2% PFA in 1× PBS (pH 7.4, 14 mM NaCl, 0.27 mM KCl, 1 mM Na_2_HPO_4_, 0.18 mM KH_2_PO_4_) followed by dehydration in ethanol series (50%, 70%, and 96%) and drying. Lampbrush chromosome maps and identification of marker structures were performed according to Callan’s monograph (Callan 1986) and the earlier study on related species from the genus *Pelophylax* (Bucci et al. 1990; Dedukh et al. 2013).

### GISH and CGH

The total genomic DNA of *P. perezi* and *P. ridibundus* was extracted from skeletal muscle tissue using Dneasy Blood and Tissue Kit (Qiagen). For CGH, the genomic DNA samples were labeled with 2´-deoxyuridine, 5´-triphosphate (dUTP) combined with biotin (*P. perezi* genome, 0.8 µg per slide) and digoxigenin (*P. ridibundus* genome, 0.8 µg per slide) using a nick translation mix [Abbott] (1 µg gDNA per reaction). Nick translation (Neusser) was performed at 17 °C for 2 h, followed by precipitation with salmon sperm DNA (ssDNA) and dissolved in a hybridization solution (50% formamide, 10% dextran sulfate, 2× SSC [sodium chloride-sodium citrate buffer, 20× SSC 3 M NaCl, 0.3 M C_6_H_5_Na_3_O_7_ · 2 H_2_O, pH 7.0], 0.04 M NaPO_4_ [sodium phosphate] buffer, 0.1% sodium dodecyl sulfate, Denhardt's reagent according to Neusser 2004).

For GISH, we used 1.2–2 µg unlabeled blocking DNA (cot10 DNA) prepared according to S1 nuclease protocol (Trifonov et al. 2009) from total genomic *P. ridibundus* DNA. Whole-genomic *P. perezi* probe was labeled by nick translation similarly to the CGH method (1 µg gDNA per reaction, 0.8 µg per slide). Further steps were identical for GISH and CGH.

Microscopic slides with chromosomal spreads were incubated with 200 ng/ml RNase solution in 2× SSC for 1 h at RT in a humid chamber, then washed three times in 2× SSC, 5 min each at RT. Solution of pepsin (0.1 mg/ml) in 0.1 M HCl was dropped on slides for 7 min RT. Slides were washed in 1× PBS for 5–10 min, dehydrated in ethanol (50%, 70%, 96%), and air dried. Chromosomes were denatured in 75% formamide at 74 °C in the water bath (GFL, Germany), followed by incubation in an ice-cold series of ethanol (70%, 80%, 96%) for 5 min each. Simultaneously, probes were denatured at 86 °C in the heating block (Grant Bio PCH1, UK) for 10 min and then put on ice. Probes were incubated on the slides covered with two 24 × 24 coverslips and glued with rubber cement (Marabu GmbH, Germany) in the humid chamber at 37 °C in the incubator (Panasonic MIR-162-PA, Japan) for 2 days. Afterward, the slides were washed in 0.2× SSC three times for 5 min each on the shaker. A blocking solution was administered (either 0.5% bovine serum albumin (BSA) in 1× PBS or 1% blocking reagent [Roche]) for 20 min. The biotin-dUTP and digoxigenin-dUTP were detected using streptavidin-AlexaFluor 488 (Invitrogen) and anti-digoxigenin-rhodamine (Invitrogen), respectively. Streptavidin-Alexa 488 and anti-digoxigenin-rhodamine were diluted in blocking solution, administered on slides, and incubated in the dark humid chamber for at least 3 h at RT. Washing was carried out in 4× SSC with 0.1% Tween pre-heated to 44 °C with shaking, three times for 5 min each. Afterward, the slides were dehydrated in ethanol series (70%, 80% 96%), air dried, and mounted in Vectashield medium containing DAPI (1.5 mg/ml) (Vector).

### Fluorescent in situ Hybridization

Labeling of the *RrS1* probe was carried out by PCR (annealing temperature 62 °C), with the genomic DNA of *P. ridibundus* serving as a template. For *RrS1* amplification, we used the forward primer 5′-AAGCCGATTTAGACAAGATTGC-3′ and the reverse primer 5′-GGCCTTTGGTTACCAAATGC-3′, following Ragghianti et al. (1995), Dedukh et al. (2019), and Chmielewska et al. (2022). The *RrS1* probe was labeled with biotin-16-dUTP (Roche).

FISH with *RrS1* probe was performed on metaphase chromosomal preparations previously used for CGH and on slides with diplotene chromosomes. CGH-treated slides were washed three times in 4× SSC with 0.1% Tween pre-heated to 44 °C in an incubator with shaking (150 rpm), leading to careful removal of the coverslips. Afterward, slides were treated according to the FISH protocol described below.

In the case of FISH with *RrS1* probe, the hybridization mixture contained 50% formamide, 10% dextran sulfate, 2× SSC, ssDNA (Sigma-Aldrich) (500–1,000 ng per slide), and PCR-labeled probe (10–50 ng per slide). Simultaneous and separate denaturations of the probe and chromosomal DNA were performed. For simultaneous denaturation, the hybridization mixture was placed directly on the chromosomal spreads, followed by heating to 75 °C for 3–5 min on the heating block (FALC, Italy). For separate denaturation, slides with chromosomal spreads were heated in 75% formamide (72 °C) for 4 min in the water bath (GFL, Germany), and placed in ice-cold ethanol (50%, 70%, 96%). The hybridization mixture was denatured in the heating block (Grant Bio PCH1, UK) at 86 °C for 7 min, then immediately put on ice for 10 min. The mixture was placed on the slides, covered with two 24 × 24 coverslips and glued with rubber cement (Marabu GmbH, Germany), and incubated in RT overnight. After hybridization, slides were washed in 4× SSC at RT to remove coverslips and then three times in 0.2× SSC heated to 47 °C (150 rpm). A blocking solution (1% blocking reagent [Roche] or 0.5% BSA in 1× PBS) was administered for 20 min. Streptavidin conjugated with AlexaFluor 488 (Invitrogen) was diluted in a blocking solution and was added for at least 3 h RT or overnight (4 °C). The slides were then washed three times in 4× SSC heated previously to 44 °C, 5 min each, then dipped in distilled water and dehydrated in ethanol (50%, 70%, 96%). Slides were mounted with Vectashield medium containing DAPI (1.5 mg/ml) (Vector). Telomeric (TTAGGG)_n_ repeat was detected on metaphase and diplotene chromosomes using a fluorescein 5-isothiocyanate-labeled peptide nucleic acid probe (DAKO, Denmark) in accordance with the providers’ instructions.

### AMD-DAPI Staining

AMD-DAPI staining was performed in accordance with Heppich et al. (1982) and Chmielewska et al. (2022), with slight changes presented below. Slides were incubated in McIlvaine's buffer pH 7.0, for 10 min, carefully drained on a paper towel, and placed in a humid chamber, where they were incubated in the darkness with actinomycin D (100 µl/slide, 0.25 mg/ml, Sigma-Aldrich) for a minimum of 20 min. Afterward, the slides were washed three times in McIlvaine's buffer for 2–3 min each, drained on paper towels, and mounted with Vectashield medium containing DAPI (1.5 mg/ml) (Vector).

### Image Processing

Chromosomes were analyzed by Zeiss Axioplan epifluorescence microscope equipped with a CCD camera and ZEISS Axio Imager.Z2 epifluorescence microscope (Zeiss, Oberkochen, Germany). Slides were scanned using 10 × objective with Metafer scanning software (MetaSystems, Altlussheim, Germany). Images of metaphase plates were recorded with a CoolCube 1 camera (MetaSystems, Altlussheim, Germany). To analyze gray-scale images, IKAROS and ISIS imaging software (MetaSystems, Altlussheim, Germany) were used. Karyotypes obtained with IKAROS were then adjusted with Krita 5.1.5 software.

## Supplementary Material

evad215_Supplementary_Data

## Data Availability

The data underlying this article are available in the article and in its online supplementary material, further inquiries can be directed to the corresponding author.
