# Supplementary material for: Cytogenetics of the Hybridogenetic Frog Pelophylax grafi and Its Parental Species Pelophylax perezi
Source: Genome Biol Evol. 2023 Nov 28;15(12):evad215. doi: 10.1093/gbe/evad215 (PMC10715190; doi:10.1093/gbe/evad215)
Supplement: evad215_Supplementary_Data [file evad215_supplementary_data.zip › Supplementary Figures 2023-10-10.pdf]

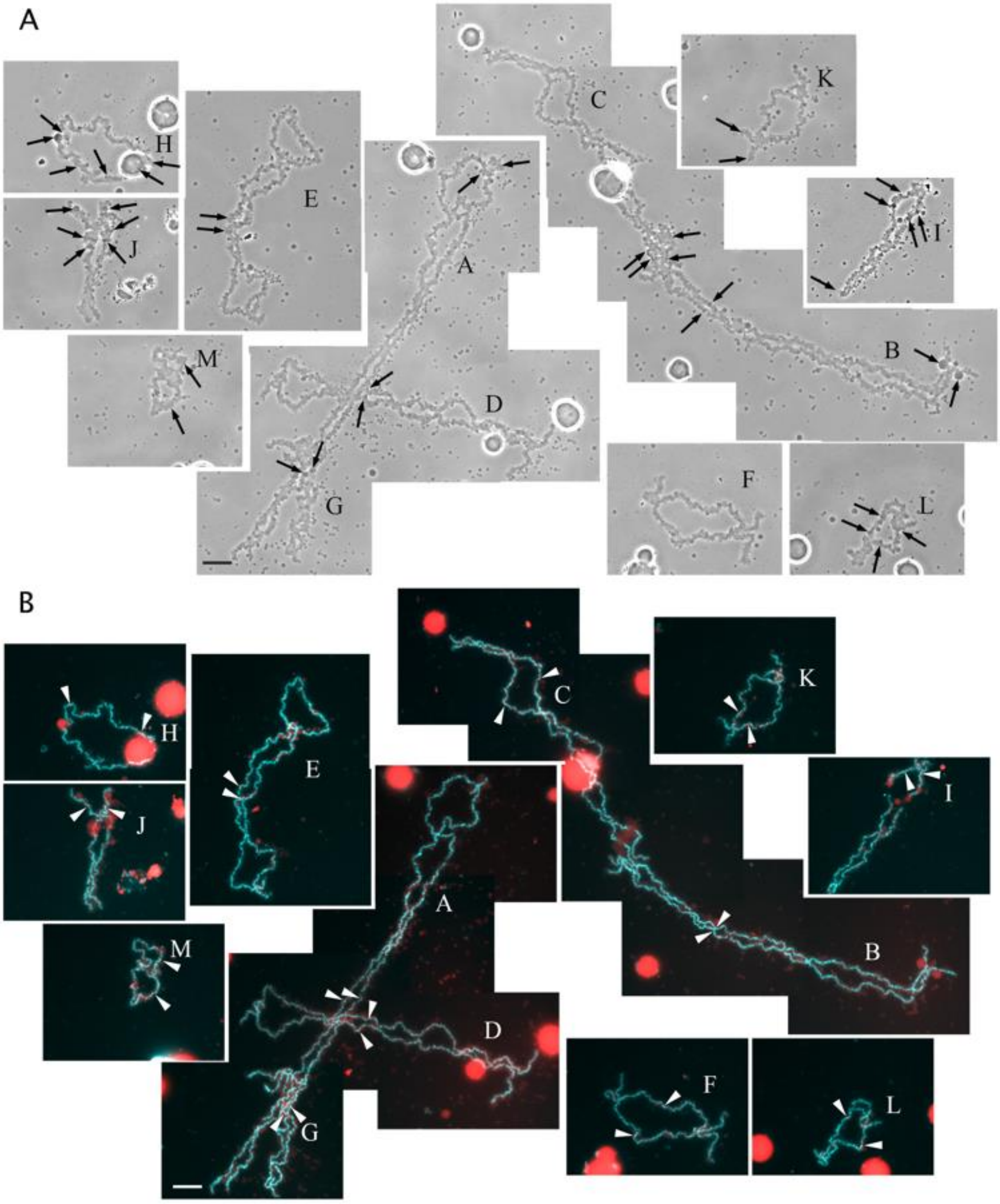

Figure S1. Identification of marker structures and pericentromeric repetitive sequence on a complete lampbrush chromosome set of *P. perezi*.

(A) Phase contrast microphotograph of a whole lampbrush chromosomal set with the identification of the most prominent marker structures (shown by arrows). (B) FISH-based mapping of pericentromeric repeat *RrS1* (indicated by arrowheads) allowed the identification of centromeric regions in lampbrush chromosomes of *P. perezi*. Since the chromosomal spread from the individual oocyte was large, several images were required to capture individual chromosomes. Lampbrush chromosomes were freely arranged to minimize a figure size. Scale bar = 10μm.

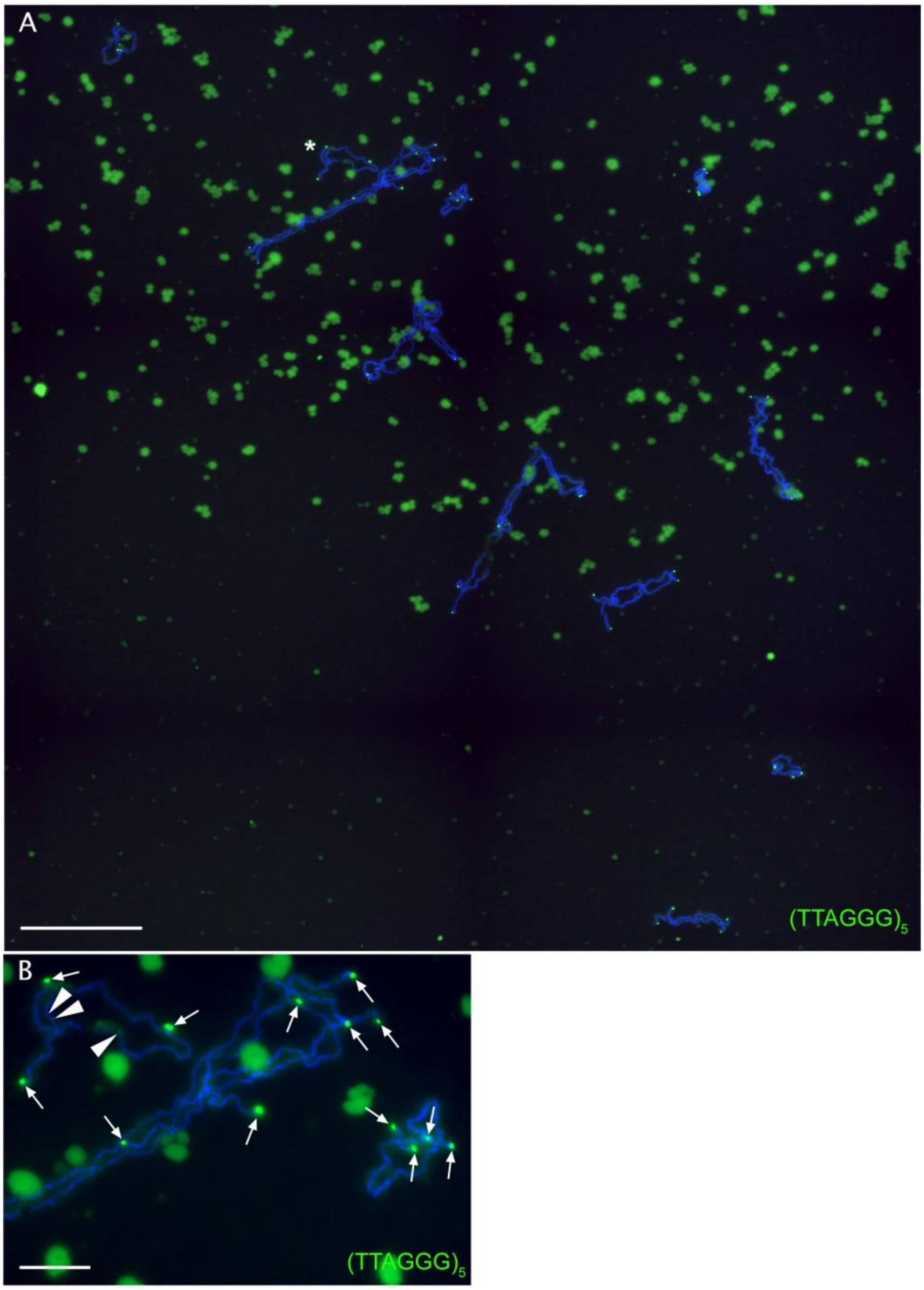

Figure S2. FISH-based mapping of (TTAGGG)-repeat sites on lampbrush chromosomes of *P. perezi*.

FISH mapping of $(TTAGGG)_n$ repeat on (A) lampbrush chromosome karyotype and (B) enlarged fragment with chromosome H with interstitial loci of $(TTAGGG)_n$ repeat, where asterisks indicates an enlarged fragment of lampbrush chromosome H. Arrows indicate telomeric blocks of $(TTAGGG)_n$ repeat; arrowheads show interstitial blocks of sequences containing $(TTAGGG)_n$ repeat in the long arm of *P. perezi* lampbrush chromosome H. As the chromosomal spread from the individual oocyte was large, six images were taken and merged into one. Scale bars: (A) 50µm, (B) 10µm.

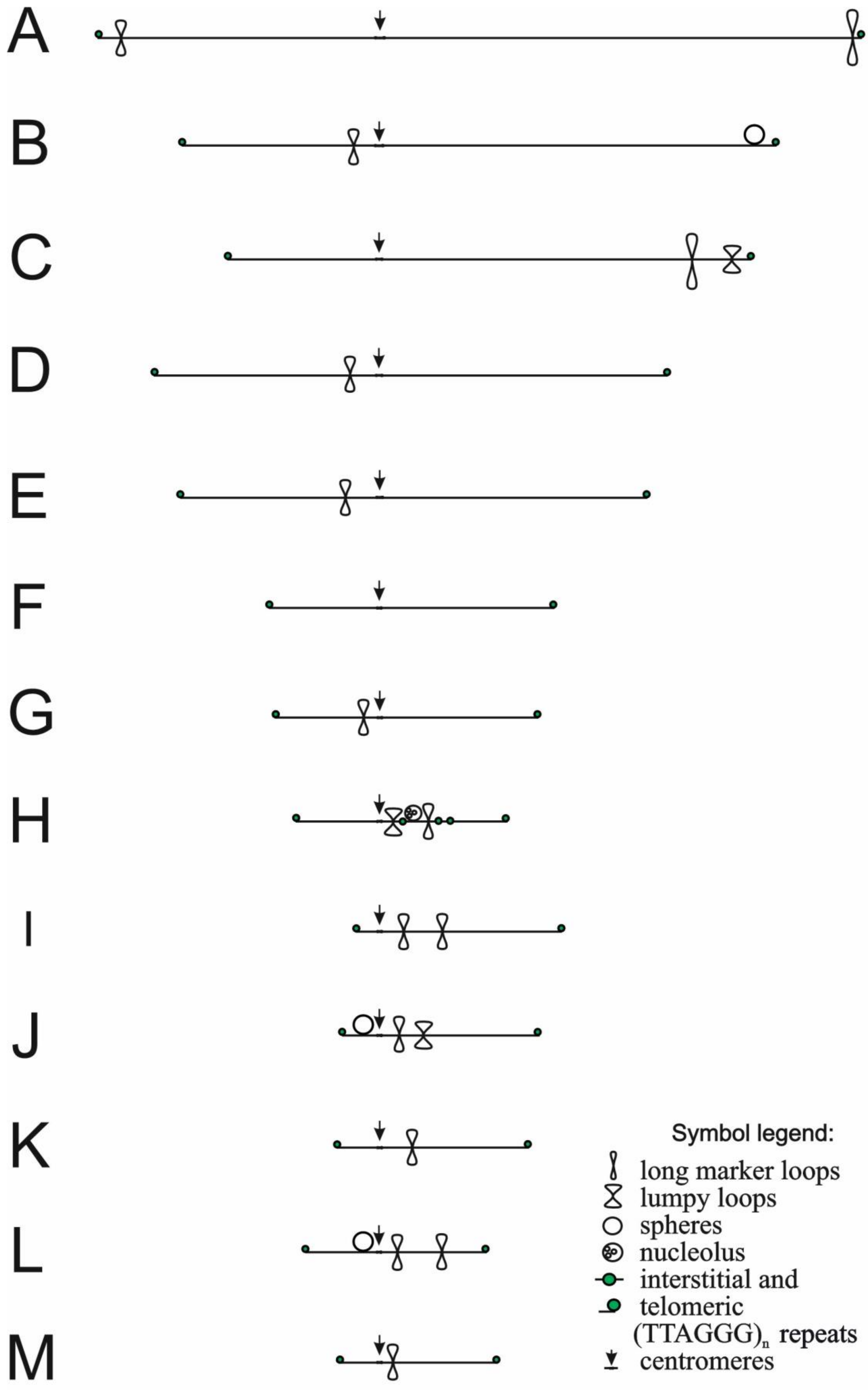
A
B
C
D
E
F
G
H
I
J
K
L
M
Symbol legend:
long marker loops
lumpy loops
spheres
nucleolus
interstitial and
telomeric
(TTAGGG)n repeats
centromeres

Figure S3. Cytological maps of lampbrush chromosomes of *P. perezi*.

Lampbrush chromosomes were arranged and lettered according to their relative length. Marker structures were mapped on chromosomes according to their comparative locations.

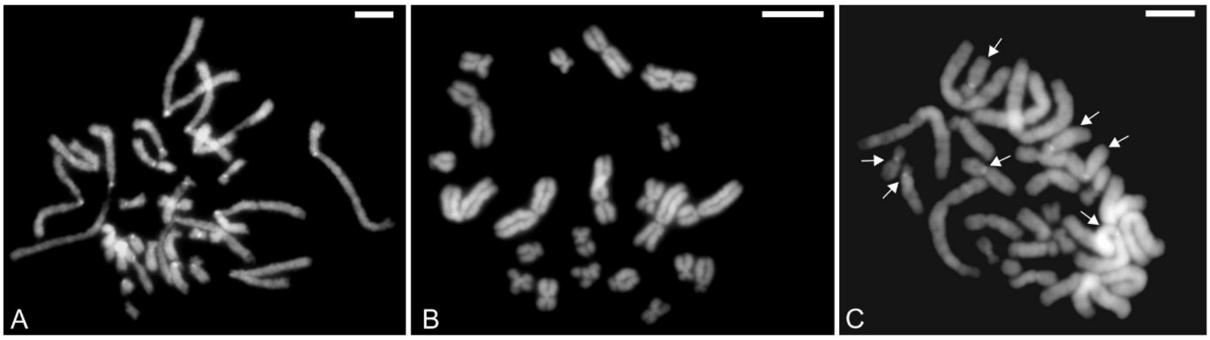

Figure S4. AMD-DAPI staining performed on metaphase plates obtained from *P. ridibundus*, *P. perezi*, and *P. grafi*.

Pelophylax ridibundus chromosomes are characterized by brighter spots in centromeric regions (A), while *P. perezi* show homogenous staining (B), which results in two distinctive sets of chromosomes in the *P. grafi* metaphase plate (C) where *P. ridibundus* chromosomes are pointed by arrows.
